# Oberhuber versus Marsh: much ado about nothing? 

**Published:** 2015

**Authors:** Amitabh Srivastava

**Affiliations:** *Pathology Department, Harvard Medical School, USA *

This issue of the journal features a spirited debate between Oberhuber and Marsh et al regarding the existence and significance of Oberhuber’s sub-classification of the Marsh III lesion into III a, b, c categories. The controversy stems from an article by Marsh and colleagues in an earlier issue of this journal where they present scanning electron microscopic (SEM) images from two cases classified as IIIc to suggest that these lesions consist of multiple crypts opening into large basins that are surrounded by concentric arrays of enterocytes. Their main contention was that Oberhuber’s category IIIa and IIIb lesions do not exist since no “atrophic” or “partially degenerate” villi were seen on the SEM examination. As Oberhuber points out in his response, included in this issue, the contention of Marsh and colleagues is supported mainly by the SEM images from two cases that were both IIIc lesions. It is certainly true that the prior study by Marsh and colleagues would have been strengthened immensely had they included a larger series of biopsies with variable degrees of villous atrophy from a well-characterized cohort of patients with celiac disease. One cannot argue with certainty regarding the absence of a particular feature or category by examining a small number of cases, no matter how sensitive the technique utilized by the observer. The debate regarding the question of Marsh category I or II lesions evolving directly into completely flat mucosa, as proposed by Marsh et al, or evolving through intermediate stages with partially degenerate villi that can be reproducibly recognized as Oberhuber’s categories IIIa or IIIb is likely to continue in the near future until a systematic large series of biopsies is examined both by routine histology and SEM examination. The Marsh classification (1) was a major paradigm shift that moved us away from the idea of some magical threshold of intraepithelial lymphocytes (IELs) or villous abnormalities that could help us confidently diagnose or rule out celiac disease. Instead, it highlighted the range of morphological possibilities that exist within patients with celiac disease. Oberhuber’s effort at introducing some standardization in reporting of duodenal biopsies in suspected celiac disease patients was a laudable one at the time it was proposed but one that has found limited use in routine diagnostic care (2).

There may still be some pathologists who believe that duodenal biopsies are the definitive diagnostic test for celiac disease but nothing could be further from the truth. The diagnosis of celiac disease rests on integration of findings from clinical history, physical examination, serological findings and morphological examination of an adequate number of duodenal biopsies. In fact, in some specific scenarios it may be possible to avoid an intestinal biopsy altogether, as advocated by the European Society of Pediatric Gastroenterology, Hepatology and Nutrition (3). It is, therefore, evident that morphology is merely one part of the puzzle in celiac disease. The two morphological features that raise the possibility of celiac disease, in routine practice, are an increased number of intraepithelial lymphocytes and abnormalities of the villous architecture. Neither is etiologically specific for celiac disease and the differential diagnosis of celiac disease mimics on biopsy, including those with villous atrophy, keeps expanding, with Olmesartan-induced enteropathy being the most recent addition to this list. On the flip side, most duodenal biopsies with normal villous architecture and increased IELs turn out not to be celiac disease but a small subset are indeed bonafide celiac disease patients who would be misdiagnosed if undue emphasis is placed upon recognition of villous atrophy.

In my opinion, the current debate surrounding the existence and significance of the Oberhuber IIIa-b lesions should be discussed in this context. Celiac disease is a challenging diagnosis with significant long-term ramifications for the patient and pathologists have an important role to play in this setting. Diagnostic categories recommended for use in daily practice should, therefore, not only be based on valid scientific observations but also on the ease of applicability and reproducibility. To this end, the Oberhuber subdivisions of the original Marsh III lesion have little to offer either in clinical practice or in fostering further advances in our understanding of the disease process. The Bucharest consensus (4) document that recently proposed the term “microscopic enteritis” for cases with normal endoscopic appearance and a spectrum of morphological abnormalities on biopsy while at the same time also suggesting a diagnostic algorithm for work up of these patients is a step in the right direction and promises to be a useful clinical guide for both gastroenterologists and pathologists*. And to pathologists still unsure about choosing between the Marsh and Oberhuber schemes, I would suggest using the Corazza (5) classification instead!

* “The editorial author was also part of the Bucharest consensus proposal on Microscopic Enteritis (reference #4)”

## Note

Please see the pages: 302-305
